# Xylem anatomy and hydraulic traits in *Vitis* grafted cuttings in view of their impact on the young grapevine decline

**DOI:** 10.3389/fpls.2022.1006835

**Published:** 2022-10-05

**Authors:** Enrico Battiston, Sara Falsini, Alessio Giovannelli, Silvia Schiff, Corrado Tani, Roberta Panaiia, Alessio Papini, Stefano Di Marco, Laura Mugnai

**Affiliations:** ^1^Sezione Patologia Vegetale ed Entomologia, Dipartimento di Scienze e Tecnologie Agrarie, Alimentari, Ambientali e Forestali, Università degli Studi di Firenze, Florence, Italy; ^2^Laboratorio di Biomorfologie, Dipartimento di Biologia, Università degli Studi di Firenze, Florence, Italy; ^3^Istituto di Ricerca Sugli Ecosistemi Terrestri, Consiglio Nazionale delle Ricerche, Sesto Fiorentino, Italy; ^4^Istituto per la Bioeconomia, Consiglio Nazionale delle Ricerche, Bologna, Italy

**Keywords:** grapevine nursery, propagation material, grafting, wounds, wood diseases

## Abstract

Grapevine grafting is an essential practice in viticulture and over the years, various bench grafting techniques have been developed to mechanize the nursery process and to increase the yield in number of viable cuttings. Bench grafting is a fundamental nursery practice that can potentially affect the quality of propagation material also in young decline associated to grapevine trunk diseases and has been recently reported to influence leaf symptoms development associated with diseases of Esca complex. The study aimed to investigate how three bench grafting methods [i.e., (i) Omega graft as mechanical technique, (ii) Whip and Tongue graft as manual technique and (iii) Full Cleft graft as semi-mechanical technique] can influence these phenomena. Specifically, the different methods were compared for their effect on the anatomical development of the grafting point and the functionality of the xylem, also considering two factors: the grapevine cultivar (Cabernet Sauvignon, Glera and Teroldego) and the scion/rootstock diameter (thin and large). Observations by light microscopy on the anatomical evolution and measurements on the xylem morphology and hydraulic traits were correlated with the grafting methods and the investigated varieties. The anatomical observations revealed that the mechanical (Omega) and semi-mechanical (Full Cleft) grafting methods have a faster callusing response while the manual technique (Whip and Tongue) has a slower but greater vascularization of the differentiated callus. Significant differences between cultivars and/or grafting types were also detected in necrotic area on the grafted tissues. Statistical analysis of the grapevine vessels suggested differences in xylem parameters between cultivars, while grafting type had no significant effects. On the other hand, the grafting type significantly affected the intrinsic growth rate. The study confirms the potential incidence of lesions and dysfunctionalities correlated with the grafting method applied, which can potentially induce grafted vine declines in vineyards due to the necrotic area detected on the grafted tissues.

## Introduction

Grafting has been used for millennia as proved by ancient literature (Mudge et al., 2009) and it is still widely exploited in horticulture and arboriculture. Since the need to counteract phylloxera damages on grapevine at the end of 19th century, grafting *Vitis vinifera* scion and American *Vitis* spp. or interspecific hybrids of *Vitis* spp. rootstock, to form one composite organism (Gautier et al., 2019) became an essential practice to establish new vineyards (Granett et al., 2001).

Over the years, several bench grafting techniques on dormant propagation material have evolved to mechanize the nursery process and to increase the yield in numbers of viable cuttings (Grohs et al., 2017), such as the Full Cleft (FC) grafting, the Whip and Tongue (W&T) grafting, and the Omega grafting. FC grafting is the easiest form of bench grafting between dormant scion and dormant rootstock and especially in the past, it was also applied in the field to graft a cutting scion on an older rooted rootstock. W&T grafting was a successful evolution of the previous method. Nowadays, among the bench grafting methods, Omega grafting is the most applied grafting technique by grapevine nurseries (Gramaje and Di Marco, 2015), combining a strong union between scion and rootstock with a high number of grafts per unit of time, and mechanizing the process with a significant reduction of the grafting cost.

Whichever grafting method is applied, a successful graft union must initially repair the mechanical damage caused by the grafting wounds. In this stage, the physical and antimicrobial barrier at the injured site consists of the accumulation of polymerized phenolic compounds such as suberin and lignin (Falsini et al., 2022). On the metabolic level, defense responses such as the production of pathogenesis-related proteins are induced and expressed by the combination of an oxidative stress and a wound-related hormone signaling (Loupit and Cookson, 2020).

Combining the tissues of two different individuals implies that their somatic tissues get involved in a process during which new vascular structures are formed and differentiated (Pina et al., 2017). The differentiation of cambial cells originates the vascular tissue, beginning with the formation of phloem vessels and then xylem vessels (Trinchera et al., 2013; Melnyk et al., 2015), and allows the connection between scion and rootstock. The mechanism responsible for such integration remains poorly understood, however, it must be significantly different for the living cells of phloem and the dead cells of xylem vessels (Melnyk, 2016).

For some scion/rootstock combinations, the relationship between the two genotypes does not always form a successful graft union and the graft interface is associated with necrosis that impact the quality of the plant, even several years after grafting (Pina and Errea, 2005). Even graft incompatibilities have been described in grapevine (Sarooshi et al., 1982). In such cases, it is still not possible to predict the longevity of the grafted plant. Moreover, at the early stage of plant development, only few visible signs can indicate a successful union (Tedesco et al., 2020).

Bench grafting is one of the fundamental nursery practices that affect the quality of the initial grapevine plant material and the global nursery yield (Waite et al., 2015). Next to the graft incompatibilities, also mechanical wounds occurring during grafting can potentially affect the functionality of the new tissues (Bavaresco and Lovisolo, 2000). Through grafting, cells are inevitably disrupted, the protective periderm layers are damaged and a rapid cicatrisation of the tissues is required to prevent water loss and especially pathogen penetration.

Viable propagules of pathogens associated with grapevine trunk diseases (GTD) have been detected on grafting machines and during the grapevine propagation process (Retief et al., 2006; Aroca et al., 2010; Gramaje and Armengol, 2011; Gramaje et al., 2011; Agustí-Brisach et al., 2013; Cardoso et al., 2013). For this reason, among the grapevine nursery operations, grafting is a critical stage as the graft wounds are inherently vulnerable to contamination with trunk pathogens (Gramaje et al., 2018). Contaminated wounds and poorly matched graft unions fail to heal properly, remain open to fungal infection, and create structural weaknesses in the finished vines (Stamp, 2001).

The occurrence of grapevine trunk pathogens in bench grafted vines has been reported by several authors (Zanzotto et al., 2001). In commercial grapevine nurseries, Fourie and Halleen (2006) found that machine grafted unions had lower GTD pathogens incidence compared to hand-grafted unions, correlating the results to the large grafting wounds created by the hand grafting method and by unsterilized hands.

The impact of the grafting practice in the grapevine decline associated to GTD was considered by some authors not only because favoring infections but also because of the influence of the physiology of the vine. Among such trunk diseases, Esca, a complex of diseases linked to fungal infections (Del Frari et al., 2019, 2022), has increased greatly over the past 20 years in Europe (Claverie et al., 2020), when also the extensive use of the mechanical grafting system, performed mainly by the omega machine, spread. Some of the diseases of the Esca complex are well known for being strongly influenced in symptoms development by the physiological reaction of the plant. Andreini et al. (2014) reported a higher percentage of leaf stripe foliar symptoms on grafted vines compared to own rooted vines. More recently, Mary et al. (2017) have investigated in depth the putative influence of grafting type on the expression of Esca leaf symptoms in mature grafted vines. The study suggests that mechanical bench grafting could be one of the factors explaining the increasing incidence of the disease in vineyard. So far, little information exists on the impact of different bench grafting methods in the physiological reaction of the vine tissues up to affecting the development of GTD symptoms.

Therefore, the purpose of the present study was to investigate (i) Omega grafting as mechanical technique, (ii) Whip and Tongue grafting as manual technique, and (iii) FC grafting as semi-mechanical technique, comparing their impact on the anatomical development of the grafting point and comparing the functional integrity of the xylem following different grafting techniques by assessing hydraulic traits in the xylem vessels of the scion. To this aim also the incidence in necrotic tissue at the grafting point and the related potential risk of the young grapevine decline in vineyard were evaluated.

## Materials and methods

### Bench grafting types

**FIGURE 1:**
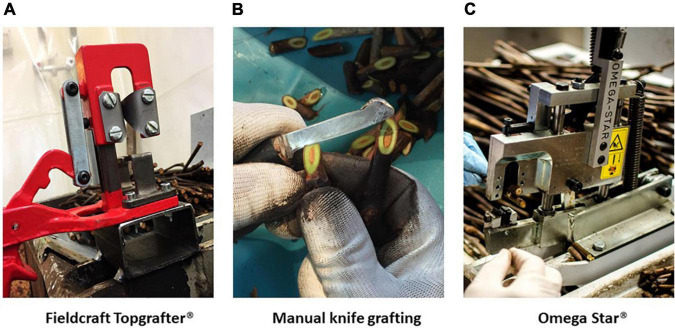
Bench grafting methods applied in nursery conditions (Vitis Rauscedo Sca, Pordenone, Italy): Fieldcraft Topgrafter^®^ instrument (Florsilva Ansaloni Srl, Bologna, Italy) fixed on a grafting table, applied as semi-mechanical method **(A)**; manual knife grafting performed by a specialized nursery operator **(B)**, applied as manual method; Omega Star^®^ machine (Fornasier Cesare & C. Snc, Pordenone, Italy) fixed on a grafting table, applied as mechanical method **(C)**.

Three methods of bench grafting were studied in the present investigation (Figure 1).

#### Full Cleft grafting

A semi-mechanical V-shaped cut was made with the Fieldcraft Topgrafter^®^ instrument (Florsilva Ansaloni Srl, Bologna, Italy) fixed on a grafting table (Figure 1A); the rootstock cutting was positioned on one side of the instrument and cut, then the scion was positioned on the other side and cut by making a reverse V-shaped cut; finally rootstock and scion were manually joined and fixed in place by taping the grafting point with a generic polyethylene cling film.

#### Whip and Tongue grafting

A manual knife cut was made by a specialized nursery operator (Figure 1B), who cut carefully the rootstock and scion, that were manually joined to form finally a strong and elongated Z-shaped junction; no taping was needed on the grafting point.

#### Omega grafting

A mechanical cut, which takes its name from the shape of the Greek letter omega, was performed by the Omega Star^®^ machine (Fornasier Cesare & C. Snc, Pordenone, Italy) fixed on a grafting table (Figure 1C); the grafting took place in two steps by first making a cut in the scion and then by inserting the rootstock cutting; no taping was needed on the grafting point.

### Plant material and growing conditions

**FIGURE 2:**
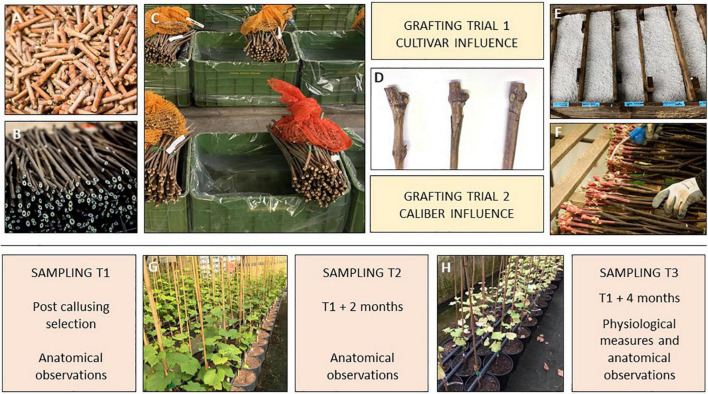
Experimental design of the bench grafting trials performed in nursery conditions (Vitis Rauscedo Sca, Pordenone, Italy): scion **(A)** and rootstock cuttings **(B)** were hydrated and disinfected prior grafting **(C)**. Propagation material was grafted according to two grafting trials **(D)**. Then callusing took place for 15 days **(E)** and finally the callused grafted cuttings were selected by discarding the non-compliant material **(F)**. Sampling T1 was performed after the post callusing selection. Sampling T2 was performed at the mid growing period **(G)** and sampling T3 was completed after the end of the growing period **(H)**.

Two types of plant material were used in the present investigation according to two different bench grafting trials (Figure 2) performed in grapevine nursery standard conditions (Vitis Rauscedo Sca, Pordenone, Italy).

#### Grafting trial 1 – Cultivar influence

The scions of three *V. vinifera* cultivars were compared according to their different susceptibility to Esca diseases (Borgo et al., 2008, 2016; Michelon et al., 2010). Glera (FEDIT 8 C.S.G. clone), Cabernet Sauvignon (169 ENTAV-INRA clone) and Teroldego (SMA 138 clone) were grafted on the rootstock *V. berlandieri* × *V. riparia* cv. Kober 5BB (FEDIT 101 C.S.G. clone) with similar caliber, variable between 7 and 10 mm. Two hundred and fifty grafts per cultivar were obtained through each bench grafting method.

#### Grafting trial 2 – Caliber influence

The scions of the *V. vinifera* cv. Glera (FEDIT 8 C.S.G. clone) were grafted on the rootstock *V. berlandieri* × *V. riparia* cv. Kober 5BB (FEDIT 101 C.S.G. clone), comparing two caliber ranges of propagation material: “thin grafted vines” obtained from scions and rootstock cuttings with caliber variable between 6 and 8 mm were compared to “thick grafted vines” obtained from scions and rootstock cuttings with caliber variable between 9 and 11 mm. Two hundred and fifty grafts per caliber range were obtained through each bench grafting method.

In both trials, the propagation material was certified according to European Directive 2005/43/EC. After harvest from the mother fields, scion and rootstock canes were cut in scion buds (5 cm) and rootstock cuttings (30 cm) and during this operation no signs of tracheomycotic infections were detected on the base and on the top of rootstock cuttings and scion buds. Prior to grafting, propagation material was hydrated by soaking in water for 12 h, and disinfected by soaking the plant material in water containing 1 mL/L of thiophanate-methyl (Enovit Metil^®^, Sipcam Italia Spa, Italy) for 30 min.

After being grafted according to each condition, grafted cuttings were waxed (Stähler Rebwachs Pro^®^, Chauvin Agro-Distribution, Sarrians, France) and placed in callusing wood box covered with sawdust and agriperlite on the top of the box. Callusing took place for 15 days at 28°C and 85% relative humidity, during which a solution containing 2.5 mL/L of thiophanate-methyl (Enovit Metil^®^, Sipcam Italia Spa, Pero (Milan), Italy) was applied as preventive treatment to control *Botrytis cinerea*. Callusing boxes were then left at environmental conditions for 10 days for the acclimatization of the callused grafted cuttings. Finally, callused plant material was removed from the boxes and selected by nursery operators in order to control: (i) the complete union between rootstock and scion and (ii) the callus uniformity. Grafted cuttings with incomplete junctions and non-uniform or hypertrophic callus were discarded. Selected grafted cuttings were planted in 6 L plastic pots with peat-rich standard soil (Huminsubstrat N17^®^, Neuhaus Italia Srl, Italy). Potted grafted vines were grown under greenhouse conditions for 4 months (mid-June to mid-October): 2 L of water per pot were supplied weekly by drip irrigation and diseases controlled by spraying the foliage with a mix of 1.5 mL/L of copper hydroxide (Kocide 2000^®^, Spiess-Urania Chemicals GmbH, Germany) and 40 mL/L of sulfur (Thiopron^®^, Upl Italia Srl, Italy) every 15 days. Three additional treatments to control grapevine pests were performed during the growing stage by applying 2 mL/L of Acetamiprid (Epik SL^®^, Sipcam Italia Spa, Pero (Milan), Italy).

### Anatomical study

Grafted samples were collected in trials 1 and 2 where the cultivar influence and the caliber effect were studied through histological observations. The sampling was carried out after callusing (T1) and 4 months after T1 (T3). Three biological repetitions (*n* = 3) for each of the three types of grafts (Omega, W&T and FC) per cultivar were selected randomly in Grafting trial 1. The same protocol was used for each of the six grafts (Omega, W&T and FC for the two calibers thin and thick) only per Glera cv. (Grafting trial 2). Graft longitudinal sections (approximately 100 μm in thickness) were cut with a Sledge G.L.S.-1 microtome (Gärtner et al., 2014).

Zeiss stereomicroscopic microscope equipped with an Optika microscope digital camera and a Leitz D.M.-R.B. Fluo Optic microscope (Wetzler, Germany) equipped with a Nikon DS-Fi3 digital camera were used for qualitative and quantitative analysis. Lugol was used for starch staining (Beccari and Mazzi, 1966).

To evaluate the most successful grafting techniques to get an efficient connection between the two bionts in the Grafting trials 1 and 2, the following qualitative and quantitative parameters were used (Tedesco et al., 2020; Table 1):

• Quality of the connection-level between scion (S) and rootstock (R) was evaluated on four categories of callogenesis. Category 4 shows a perfect connection between bionts, where callus covered the space between S and R and the graft line is practically unrecognizable. Category 3 shows a few structural imperfections in the graft border where the connection between the two bionts is almost complete. Category 2 is characterized by many discontinuities (wood or bark) in the border and in category 1 S and R are unattached and an intermediate space is clearly observed.
• Anatomical part of bionts involved in grafting connection which means parenchymal tissue or cambium of S and R, respectively.
• Internal callus (between bionts) differentiation grade.
• External callus differentiation grade or the callus formed around the graft union (outside bionts).
• Occurrence of necrosis areas in S or R, or in both of them.
• Space between bionts.

**TABLE 1:** Quantitative parameters used for grafting techniques evaluation.

Callogenesis	
1	Broken/unattached union
2	Lots of discontinuities
3	Few structural imperfections
4	Perfect union
**Bionts involved in graft connection**	
a	Scion parenchymatic rays
b	Scion cambium
c	Rootstock parenchymatic rays
d	Rootstock cambium
**Callus type**	
0	Non-vascular
1	Differentiated without meristemoids
2	Differentiated with meristemoids
–1	Necrotized
**External callus**	
0	Non-vascular
1	Differentiated
**Necrosis**	
S	Scion
R	Rootstock
np	Not present
**Space between bionts**	
Yes	Presence
No	Absence

### Necrosis area measurements

Necrosis areas were considered as degenerated tissues that appeared dark colored compared to the healthy tissue. These regions were measured by analyzing the callus growth at a quarter of the sample diameter, where the parenchymatic rays and cambium of the two bionts were observed. The necrosis areas were measured with Image J software 1.47v.

### Xylem morphology and hydraulic traits

In both trials, sections 2–3 cm long were cut from 1-year-old stems during dormancy. In trial 1, the 10th internode was selected for each combination of cultivars and grafting type while in trial 2, the 6th internode was sampled from Glera grafted cuttings. All the stem samples were placed in a 50:50 (v/v) mixture of ethanol and water and stored at 5°C. The stem sections were then fixed through ice on a Peltier plate, and transverse sections of 10–12 μm thickness cut using a rotary microtome (Haworth et al., 2017). The sections were stained with a solution of 0.04% safranin, 0.15% astrablue and 2% acetic acid in distilled water, and permanently fixed with a histological mounting medium (Eukitt, Bioptica, Milan, Italy). Anatomical observations were made with a Nikon Eclipse 800E light microscope and images recorded with a Nikon DS-Fi2 camera attached to the microscope (Nikon Corporation, Tokyo, Japan). Each section was divided into lateral (L) and dorso/ventral (DV) sectors as proposed by Pouzoulet et al. (2020) and four orthogonal subsectors were defined (two L and DV sectors for each stem cross-section respectively). The subsectors contained from four to six xylem areas (Xy) each one delimited by two consecutive parenchymatic bands (lateral), phloem (outer) and pith (inner). Thus, Xy was exclusively composed of xylem elements (fibers and vessels). Digital images of each Xy were then analyzed using the computer program Nikon Nis-Elements^®^ software and the following parameters measured: Xy area (μm^2^); lumen vessels area (VA, μm^2^); vessel density (Vd, mm^–2^). The diameter of each vessel (*d*_*v*_, μm) was calculated as the diameter of a circle with an area equivalent to VA.

The hydraulic weighted vessel diameter *Dh* (μm) was calculated following Sperry and Saliendra (1994) as:


(1) $$D\,h=\frac{\Sigma \,d_{v}^{5}}{\Sigma \,d_{v}^{4}}$$

Calculation of *Dh* incorporates the disproportionate contribution of large vessels to total flow and gives the average diameter needed for a given vessel density to result in the theoretical hydraulic conductivity for that stem Tyree et al. (2002).

The theoretical specific xylem hydraulic conductivity (*K*_st_) was calculated following Santiago et al. (2004) using the Hagen–Poiseuille equation for ideal capillaries assuming laminar flow:


(2) $$k_{s\,t}=\left(\frac{\pi \,\rho }{1.28\,\eta \,A_{i}}\right)\,\Sigma \,d_{v}^{4}$$

where ρ is the density of water (998.2 Kg m^–3^ at 20°C); η is the viscosity of water (1.002 × 10^–9^ MPa s at 20°C), and *A*_*i*_ is the area of the image analyzed (m^2^).

### Plant growth

In the trial 1, 15 grafting were randomly selected for each cultivar and grafting type combination and biweekly measured (height and number of internodes) from day of the year (DOY) 182 to 281 (July 1st to October 8th). The relative growth rate (RGR) was calculated for internode number and plant height fitting biweekly measurements by Logistic functions at three parameters as follow:


(3) $$y=\frac{k\cdot y_{0}}{y_{0}+\left(k-y_{0}\right)\,e^{-r\,t}}$$

where *y* corresponds to actual value of the measure, *y*_0_, initial value of measure (*t*_0_), *r*, intrinsic growth rate (1/time), *k*, carrying capacity (maximum value of the measure), *t*, time. The parametrization of RGR were determined using *growth rates* R statistical packages software R 0.8.2 (Petzoldt, 2020).

### Statistical analysis

Hydraulic traits were analyzed on five biological replicates (*n* = 5) while necrosis percentage was analyzed on three biological replicates (*n* = 3). For growth parametrization there were fifteen biological replicates per cultivar and grafting type (*n* = 15). Data were checked for normal distribution (D’Agostino–Pearson’s χ^2^ test) and homogeneity of variance (Levene tests). Percentages were transformed by arcsin function and normal distribution verified before analyses. In both trials, the effects of the cultivars and grafting type were assessed with two-way ANOVA. *Post hoc* analysis was conducted using Bonferroni test. Correlation was evaluated by performing Pearson’s test. All analyses were executed using dplyr, multcomp and car R statistical packages software R 4.0.2 (R Core Team, 2020).

## Results

### Grafting trial 1 – Cultivar influence

#### Anatomical observations

##### Glera (FEDIT 8 C.S.G. clone) scion on the rootstock *Vitis berlandieri* × *Vitis riparia* cv. Kober 5BB (FEDIT 101 C.S.G clone)

**FIGURE 3:**
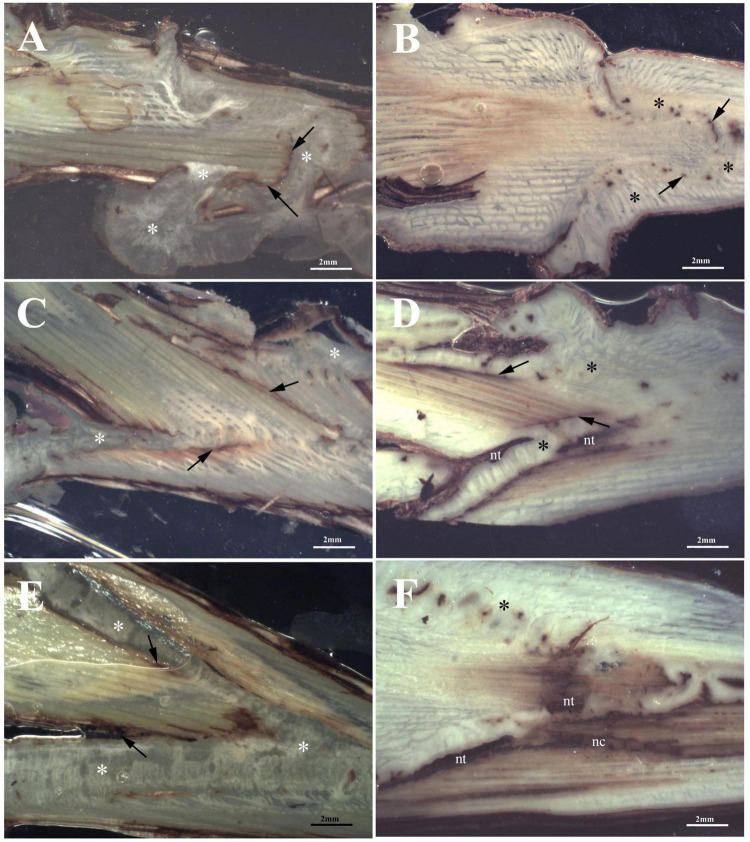
Stereoscopic images of graft longitudinal sections of Glera scion onto K5BB rootstock, at T1 **(A,C,E)** and T3 **(B,D,F)**. Callus formation in different graft union shapes: Omega **(A,B)**, W&T **(C,D)**, and FC **(E,F)**. *, Non differentiated callus; *Differentiated callus; nt, necrotic tissue; nc, necrotic callus; black arrow, discontinuity.

At T1, Omega grafting (Figure 3A) showed the highest callus formation (callogenesis = 3) in comparison to W&T (Figure 3C) and FC (Figure 3E) grafting (callogenesis = 2) (Table 2). In all three grafted cuttings the different tissues of bionts such as parenchymatic rays and cambium either of S or R, were almost equally involved since the beginning. Moreover, the callus between bionts appeared not structured and without necrosis (Supplementary Figures S1A,C,E) as well as the external one, as shown in Supplementary Figure S2A. As shown in Figure 3A, the space between S and R was evident and filled by callus, which ensured the continuity between the two bionts.

**TABLE 2:** Quantitative evaluation (see Table 1) of Glera, Cabernet Sauvignon, Teroldego scion onto K5BB rootstock comparing three different techniques (Omega, WT, and FC) at T1 and T3.

Cultivar	Survey time	Grafting techniques	Callogenesis	Bionts involved in the connections	Type of callus	External callus	Necrosis	Space between S and R	Ranking
Glera		Omega	3	3a, 3b, 3c, 3d	0	0	np	Yes	1
	**T1**	W&T	2	3a, 3b, 3c, 3d	0	0	np	Yes	2
		FC	2	3a, 2b, 3c, 3d	0	0	np	Yes	3
		Omega	4	3a, 3b, 3c, 3d	1	1	S, R	Yes	1
	**T3**	W&T	3	2a, 3b, 2c, 3d	1	1	S, R	Yes	2
		FC	3	3a, 3b, 3c, 3d	1; –1	1	S, R	Yes	3
Cabernet Sauvignon	**T1**	Omega	3	3a, 3b, 3c, 3d	0	0	np	Yes	1
		W&T	2	3a, 3b, 3c, 3d	0	0	np	Yes	2
		FC	3	3a, 3b, 3c, 3d	0	0	np	Yes	1
	**T3**	Omega	4	3a, 3b, 3c, 3d	1	1	S, R	No	2
		W&T	4	3a, 3b, 3c, 3d	1	1	S, R	No	1
		FC	2	3a, 3b, 3c, 3d	1; –1	1	S, R	Yes	3
Teroldego	**T1**	Omega	2	1a, 1b, 3c, 3d	0	0	np	Yes	2
		W&T	3	1a, 3b, 3c, 3d	0	0	np	Yes	1
		FC	2	2a, 2b, 3c, 3d	0	0	np	Yes	2
	**T3**	Omega	3	3a, 3b, 3c, 3d	2; –1	1	S	Yes	2
		W&T	4	3a, 3b, 3c, 3d	2; –1	1	S	Yes	1
		FC	2	3a, 3b, 3c, 3d	2; –1	1	S, R	Yes	3

At T3 Omega grafting (Figure 3B) still showed the highest callus formation (callogenesis = 4) in comparison to W&T (Figure 3D and Supplementary Figure S1D) and FC (Figure 3F) grafting (callogenesis = 3) (Table 2). As in T1, in all three grafted cuttings the different tissues of bionts such as parenchymatic ray and cambium of either S or R continued to be involved. Moreover, at T3 the callus derived from the parenchymal and cambial cells, differentiated into wood vessels in all the three grafted cuttings. Newly formed xylem vessels were documented in Supplementary Figure S2B. All the three grafted cuttings were characterized by necrosis of the S and R, respectively, which was not observed at T1. Furthermore, at this stage the FC grafting showed the external callus more necrotized (Supplementary Figure S1F) than the other grafted cuttings. The space between S and R was evident mainly in the W&T and FC grafting where it was filled by callus but some discontinuities were evident and delimited by necrotic tissues.

##### Cabernet Sauvignon (169 ENTAV-INRA clone) scion on the rootstock *Vitis berlandieri* × *Vitis riparia* cv. Kober 5BB (FEDIT 101 C.S.G clone)

**FIGURE 4:**
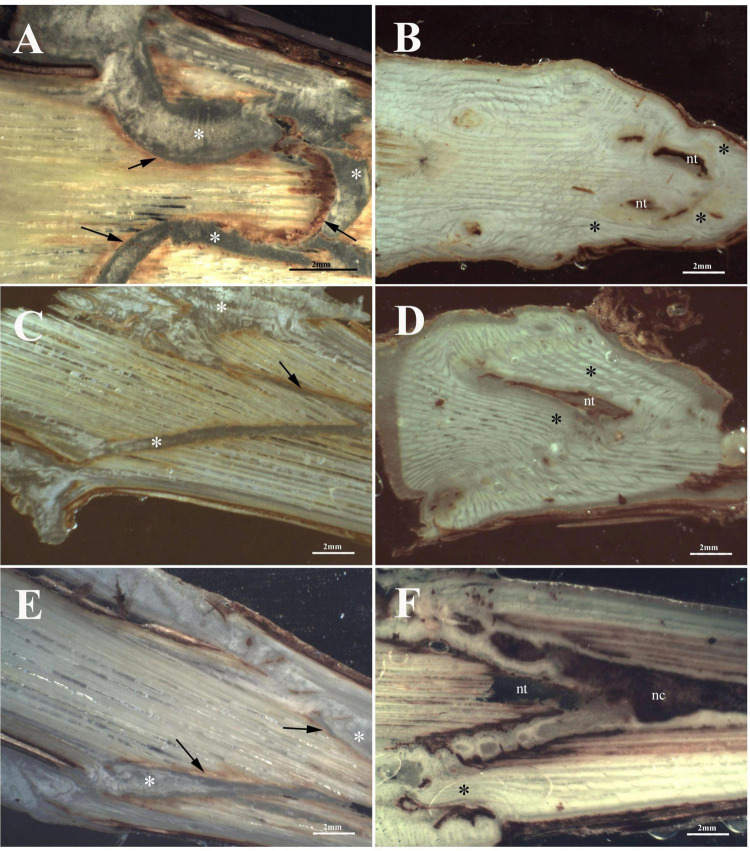
Graft longitudinal sections of Cabernet Sauvignon scion onto K5BB rootstock, at T1 **(A,C,E)** and T3 **(B,D,F)**. Callus formation in different graft union shapes: Omega **(A,B)**, W&T **(C,D),** and FC **(E,F)**. *, Non differentiated callus; *Differentiated callus; nt, necrotic tissue; nc, necrotic callus; black arrow, discontinuity.

At T1, concerning callus proliferation, Omega and FC grafting showed a higher score (callogenesis = 3) than W&T grafting (callogenesis = 2) (Table 2). In all three grafted cuttings the different tissues of bionts such as parenchymatous ray and cambium either of S or R were almost equally involved in the callus generation since the beginning. Moreover, the callus between bionts appeared not structured and without necrosis (Figures 4A,C,E) as did the external one. The space between S and R was evident in all three grafting and was filled by callus, which ensured the tissue continuity between bionts. At T1 the highest production of external callus around the grafting samples (Supplementary Figure S3A) was observed in the FC grafting compared to Omega and W&T grafting (Supplementary Figures S3C,E). As well as in Glera (Grafting trial 1), the callus filling the space between R and S was pearly white, hyperhydric and rich in calcium oxalate crystals (Supplementary Figure S4A).

At T3, both Omega (Figure 4B) and W&T (Figure 4D) grafting showed successful connections between bionts. Only some discontinuities by the bark tissue were still observed. In particular, Omega grafting showed necrosis in both S and R (Supplementary Figure S3B) as well as in W&T (Supplementary Figure S3D). FC grafting (Figure 4F and Supplementary Figures S3F,S5A–F) appeared the worst in terms of graft union because necrotized areas were frequently found in both bionts and in the internal callus. In general, at T3 the callus was well structured and xylem vessels were recognizable among parenchymatous tissue rich in starch (Supplementary Figure S4B).

##### Teroldego (SMA 138 clone) scion on the rootstock *Vitis berlandieri* × *Vitis riparia* cv. Kober 5BB (FEDIT 101 C.S.G clone)

**FIGURE 5:**
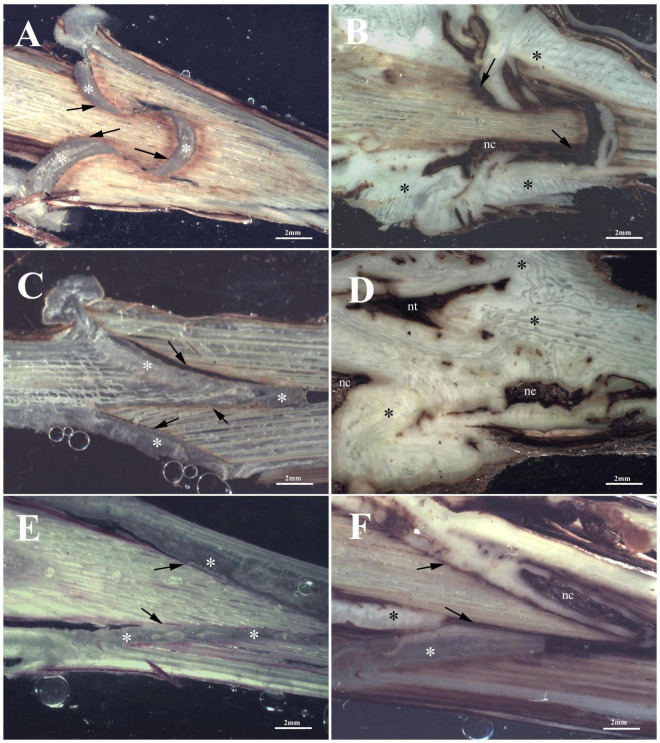
Graft longitudinal sections of Teroldego scion onto K5BB rootstock, at T1 **(A,C,E)** and T3 **(B,D,F)**. Callus formation in different graft union shapes: Omega **(A,B)**, W&T **(C,D)**, and FC **(E,F)**. *, Non differentiated callus; *Differentiated callus; nt, necrotic tissue; nc, necrotic callus; black arrow, discontinuity.

At T1, W&T showed the best callogenesis which was mostly produced by both rootstock’s cambium and parenchymatic rays and only in part by scion cambium (Figure 5C). In FC grafting, both S and R were involved in the initial callogenesis (Figure 5E) although the highest level of callus production was due to rootstock (Table 2). Omega grafting showed a callogenesis level similar to FC grafting (lower than W&T grafting) where the R contribution was the most significant in graft union formation (Table 2). Moreover, in all three grafting methods, the callus between bionts was not differentiated and no necrotic areas in the internal callus as well as in the external one was observed (Figures 5A,C,E). As in Glera and Cabernet Sauvignon grafted cuttings, the callus filling the space between R and S was pearly white and hyperhydric. In particular, Supplementary Figure S6A showed an example of the callus formed by parenchymatous tissue completely filling the spaces between the two components connecting the S and the R.

At T3, W&T still showed the highest callus production (callogenesis = 4) (Figure 5D), followed by Omega (callogenesis = 3) (Figure 5B) and FC (callogenesis = 2) (Figure 5F) with some bark discontinuities between S and R. At T3, in all three grafted cuttings, the different tissues of the bionts, such as parenchymatic ray and cambium of both S and R were involved in callus generation (Table 2), in comparison with T1. Moreover, in all three grafted cuttings the callus was characterized by meristemoids with xylem vessels in differentiation (Supplementary Figure S6B). This feature seems to belong exclusively to Teroldego cv. In both Omega and W&T grafting, necrotic areas were observed especially in the S, whereas FC grafting showed necrosis in both S and R. The space between S and R was mainly evident in Omega and FC grafting where space was filled by callus but some discontinuities were still observed especially in the scion border, which was characterized by necrotic tissue.

#### Necrosis area statistical analysis

Statistical analysis on the necrosis percentage area indicated that significant differences were observed between cvs and/or grafting type (Table 3). In particular, regarding Cabernet Sauvignon, W&T grafting showed an amount of necrotic tissue (20.2 ± 3.1%) similar to Omega grafting (21.7 ± 4.2%). FC grafting necrosis value was significantly higher (43.8 ± 1%). A similar trend was evidenced in cv. Teroldego where the values of necrosis between W&T and Omega grafted cuttings (30.9 ± 7.6% and 37.3 ± 4.2%, respectively) were similar but significantly different from FC (63 ± 0.9%). On the contrary, considering cv. Glera, FC and Omega grafted cuttings (31.1 ± 1.4% and 36.1 ± 3%, respectively) showed the lowest necrosis value in comparison to W&T grafting (56.6 ± 1.8%).

**TABLE 3:** Different a two-way ANOVA was used to assess any significant differences between cvs and/or grafting type.

Cultivar	Grafting type	Necrosis (%)	Vd (mm^–2^)	VA_f_ (%)	Dh (μ m)	*K*st (Kg MPa^–1^ s^–1^ m^–1^)
	W&T	56.6 ± 1.8e	101 ± 15.1ab	23.7 ± 3.7	31.6 ± 3.1	34.5 ± 7.4
Glera	Omega	36.1 ± 3cd	137 ± 30.3a	22.1 ± 1.6	25.7 ± 2.6	32.1 ± 14.5
	FC	31.1 ± 1.4bc	125 ± 18.3ab	21.5 ± 2.8	27.4 ± 2.2	39.9 ± 11.1
	W&T	30.9 ± 7.6bc	130 ± 24.9ab	21 ± 3.7	26.3 ± 4.1	41.1 ± 12.7
Teroldego	Omega	37.3 ± 4.2cd	110 ± 21.2ab	17.8 ± 1.4	26.4 ± 2.7	31 ± 6.8
	FC	63 ± 0.9e	125 ± 29.2ab	20.2 ± 2.2	25.7 ± 4.1	31.5 ± 7.5
	W&T	20.2 ± 3.1a	97.9 ± 4.7ab	18.6 ± 1.3	27.8 ± 2.8	28.9 ± 9.2
Cabernet Sauvignon	Omega	21.7 ± 4.2ab	112 ± 23.7ab	18.7 ± 2.9	27 ± 3.6	26 ± 7.2
	FC	43.8 ± 1d	85.2 ± 6.5b	18.6 ± 3.2	30.9 ± 3	33.8 ± 14.6

**Two-way ANOVA**	**Df**	***F*-value**				

Cultivars	2	45.72***	5.813**	8.156**	2.591^ns^	1.38^ns^
Grafting type	2	36.88***	0.99^ns^	1.33^ns^	1.928^ns^	1.236^ns^
Cultivars × Grafting type	4	55.39***	3.036*	0.793^ns^	2.349^ns^	0.821^ns^
Residuals	36					

Different letters indicate significant differences between datasets based upon a one-way ANOVA with Bonferroni post hoc test. Necrosis percentages were transformed by arcosin function and normal distribution verified before two-way ANOVA.

*P < 0.05, **P < 0.01, and ***P < 0.001.

#### Physiological measurements

Results are presented according to each cultivar (Glera, Cabernet Sauvignon, Teroldego) grafted on the common rootstock Kober 5BB and the applied bench grafting method (Omega, FC, W&T). No plant material was discarded by this operation; however, the callus feature was clearly different according to each bench grafting type, especially on cv. Glera: homogeneous callus in Omega grafted cuttings, homogeneous and hypertrophic callus in W&T grafted cuttings and less complete and hypertrophic callus in FC grafted cuttings.

##### Xylem morphology and hydraulic traits

Analysis of the grapevine vessels suggest differences in xylem traits between the three cultivars whilst grafting type did not have significant effects for all the considered traits (Table 3). The cultivar Cabernet Sauvignon exhibited significant lower xylem vessel density than Glera and Teroldego (in average 98.5 mm^–2^ vs. 120 and 121 mm^–2^ respectively). However, the significant interaction (*P* < 0.01) indicates that the Vd was affected by grafting type in relation to cultivars. Thus, Vd was significantly higher in the combination Glera-Omega than Cabernet Sauvignon-FC (137 ± 30.3 mm^–2^ and 85.2 ± 6.5 mm^–2^ respectively). Like Vd, the ratio vessel area: xylem area was significantly affected by cultivars. Glera had significant higher VA_f_ than Teroldego and Cabernet Sauvignon (in average 22.4% vs. 19.7 and 18.6% respectively). On the contrary, hydraulic traits (Dh and Kst) were not affected by cultivar and grafting type. Dh ranged from 31.6 ± 3.1 and 25.7 ± 2.6 μm and Kst from 41.1 ± 12.7 and 26 ± 7.2 Kg Mpa^–1^ s^–1^ m^–1^. At the whole, a high vessel density determined a reduction of Dh (Table 4). Correlation analyses evidenced a positive and significant relationship between VD and Dh (*P* = 0.02).

**TABLE 4:** Correlation between the cultivars evaluated by the Pearson’s test.

	Vd	VA_f_	Dh	*K*st
Vd	1.00			
VA_f_	0.25	1.00		
Dh	–0.74*	0.37	1.00	
*K*st	–0.03	0.12	0.07	1.00

*P < 0.05.

##### Plant growth

**FIGURE 6:**
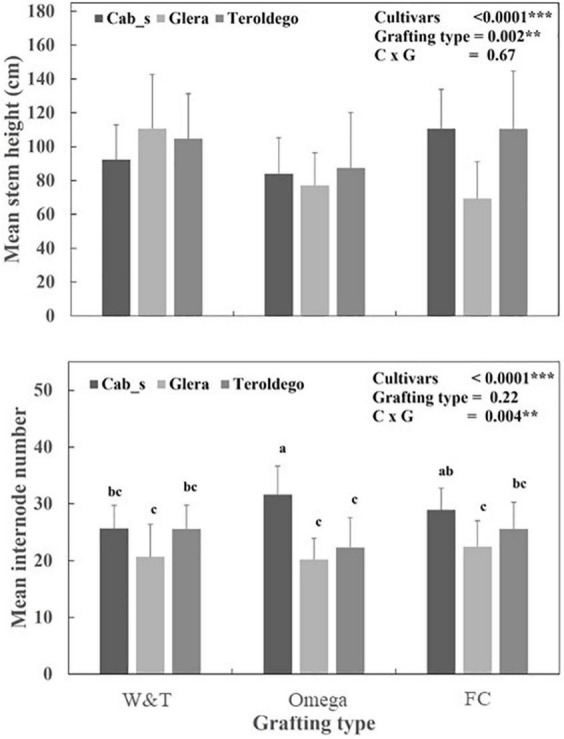
Mean value and standard deviation for mean stem height and internode numbers measured in Teroldego, Glera and Cabernet Sauvignon cultivars scion onto K5BB rootstock with Whip & Tongue (W&T), Omega, and Full Cleft (FC). Two-way ANOVA was used to assess any significant differences between treatment and/or cvs. Different letters indicate significant differences between datasets based upon a one-way ANOVA with Bonferroni *post hoc* test. **P < 0.01,***P < 0.001.

Comparison of the parameters of the Logistic function revealed different patterns of stem growth dynamics relatively to cultivars for internode number and cultivar/grafting type for plant height measurements (Figure 6, Supplementary Table S1 and Table 5). *R*^2^ were higher than 0.98 for all fitted curves.

**TABLE 5:** Parametrization of plant height by Logistic model.

Cultivar	Grafting type	Logistic model		
		*K*	y_0_	*r*
	Omega	70.5 ± 19.3	4.9*10^–9^	0.13 ± 0.02
Glera	FC	81.8 ± 21.8	1.7*10^–6^	0.12 ± 0.04
	W&T	79.6 ± 32.3	8.3*10^–6^	0.11 ± 0.03
	Omega	87.7 ± 32.7	7.5*10^–7^	0.13 ± 0.03
Teroldego	FC	112 ± 34.9	3.6*10^–6^	0.12 ± 0.03
	W&T	108 ± 27.4	2.3*10^–6^	0.1 ± 0.03
	Omega	85.3 ± 20.9	8*10^–9^	0.14 ± 0.03
Cabernet Sauvignon	FC	114 ± 23.2	1.1*10^–7^	0.11 ± 0.02
	W&T	93.9 ± 20.4	1*10^–7^	0.13 ± 0.02

**Two-way ANOVA**	**Df**	***F*-value**		

Cultivars	2	11.434***	0.948^ns^	0.955^ns^
Grafting type	2	7.405***	0.95^ns^	7.153**
Cultivars × Grafting type	4	0.737^ns^	0.754^ns^	1.149^ns^

*P < 0.05, **P < 0.01, and ***P < 0.001. ns, not significant.

As shown in Figure 6, the three cultivars significantly differed for *K* (*P* < 0.0001). Cabernet Sauvignon showed a higher *K* and *r* coefficients than Teroldego and Glera (30.2 vs. 25.8 and 22.7 number of internodes respectively). However, the significant interaction (*P* < 0.01) for *K* coefficient indicates that the final number of internodes per plants was affected by grafting type within each cultivar. The Omega and FC grafting induced the higher number of internodes in Cabernet Sauvignon (in average more than 30 internodes each) and the lower in Glera (in average 23 internodes). Teroldego and Cabernet Sauvignon had significantly higher intrinsic growth rate than Glera (0.062–0.063 DOY^–1^ vs. 0.056 DOY^–1^ respectively, *P* < 0.0001).

As shown in Table 5, the K coefficient was significantly affected by cultivars (*P* < 0.0001) and grafting type (*P* = 0.0001). Teroldego and Cabernet Sauvignon showed the higher *K* than Glera (in average 102.6, 97.6, and 77.3 cm) whilst FC determined significant high *K* coefficients than Omega (102.6 vs. 81.2 cm respectively). The intrinsic growth rate, *r*, was significantly affected by grafting type (*P* = 0.001). Omega grafting determined a higher *r* for plant height than FC and W&T.

## Grafting trial 2 – caliber influence

### Anatomical observations

*Vitis* vinifera L. cv. Glera (FEDIT 8 C.S.G. clone) was grafted on the rootstock *V. berlandieri* × *V. riparia* cv. Kober 5BB (FEDIT 101 C.S.G. clone).

**FIGURE 7:**
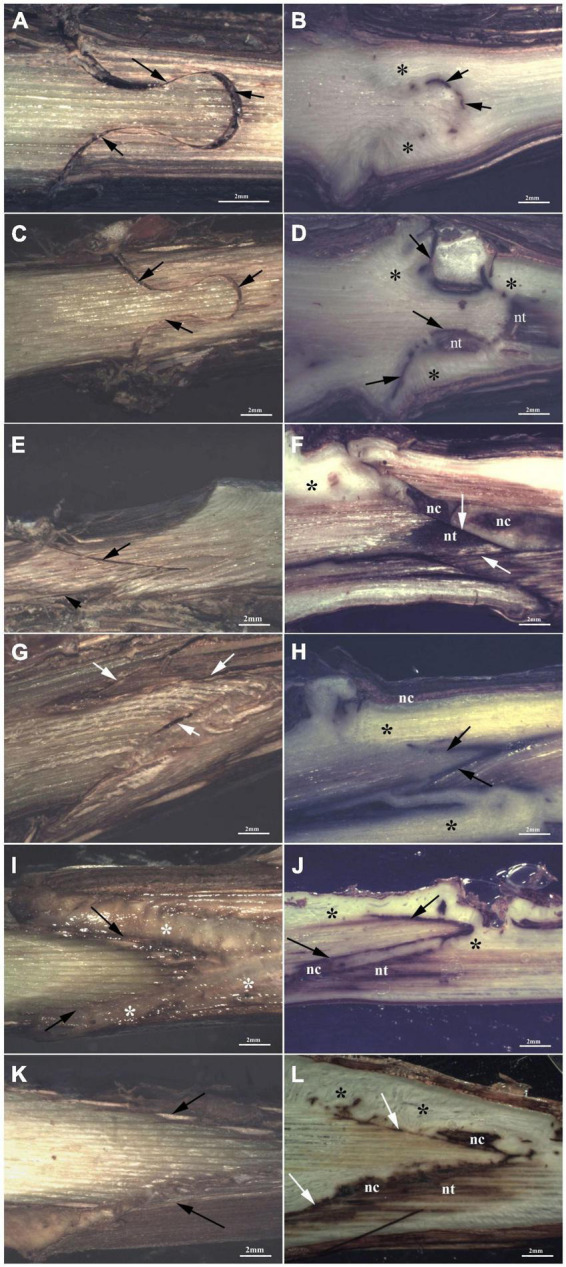
Graft longitudinal sections of Glera scion onto K5BB rootstock, at T1 **(A,C,E,G,I,K)** and T3 **(B,D,F,H,J,L)**. Callus formation in different graft union shapes: Omega-thin **(A,B)**, Omega-thick **(C,D)**, W&T-thin **(E,F)**, W&T-thick **(G,H)**, FC-thin **(I,J)**, FC-thick (**K**,**L**). *, Non differentiated callus; *Differentiated callus; nt, necrotic tissue; nc, necrotic callus; black arrow, discontinuity.

At T1, FC-thin showed the highest connection between bionts (Figure 7I) in comparison to other grafting techniques (Figures 7A,C,E,G,K) even though several discontinuities were observed. Omega thin and thick (Figures 7A,C) and W&T thick (Figure 7G) grafted cuttings showed unattached unions with evident empty spaces. In particular, the callus production in FC-thin grafting was almost due to parenchymatic rays of both R and S and only in part by R’s cambium (Table 6). In all techniques, a characteristic found common was the parenchymatic rays contribution of both bionts in grafting connection (Table 6). A peculiarity was observed in the callus of FC thin which was already differentiated at T1, as shown in Supplementary Figure S8A. Necrotic areas were found only in S and R in W&T thin and thick (Supplementary Figures S7E,G) compared to the other grafted cuttings (Supplementary Figures S7A,C,K).

**TABLE 6:** Quantitative evaluation of Glera.

	Grafting type	Callogenesis	Bionts involved in the connections	Type of callus	External callus	Necrosis	Space between S and R	Ranking
T1	Omega–Thin	1	2a, 1b, 1d, 1c	0	0	np	Yes	3
	Omega–Thick	1	3a, 2c, 2d	0	0	np	Yes	2
	W&T–Thin	1	1a, 2b, 3c	0	0	S e R	No	5
	W&T–Thick	1	3a, 1b, 2c	0	0	S e R	Yes	5
	FC–Thin	2	3a, 3c, 2d	1	0	np	No	1
	FC–Thick	1	3a, 2c, 2d	0	0	np	No	2
T3	Omega–Thin	4	3a, 3b, 3c, 3d	1	1	S	No	1
	Omega–Thick	3	3a, 3b, 3c, 3d	2	1	S and R	Yes	2
	W&T–Thin	2	3a, 3b, 3c,3d	1; –1	1	S and R	Yes	4
	W&T–Thick	3	3a, 3b, 3c, 3d	1;–1	1	R	Yes	3
	FC–Thin	3	3a, 3b, 3c, 3d	1; –1	1	R	Yes	3
	FC–Thick	3	3a, 3b, 3c, 3d	1; –1	1	R	No	3

See Table 1 of Glera scion onto K5BB rootstock comparing six different techniques (Omega, W&T, FC per two calibers) after callusing (T1) and 4 months after T1 (T3).

At T3, Omega thin showed the best connection between bionts (Figure 7B) where the callus completely filled the space and the graft border was unrecognizable. The connection was not only external (Supplementary Figure S7D) but also involved parenchymatic rays and cambium activation excluding the pith. The proliferation of parenchymatic rays and cambium to form callus is a characteristic found in all grafting techniques (Figures 7D,F,H,J,L and Supplementary Figures S7F,H–J,L) not only in Grafting trial 2 but also in the trial 1 (Supplementary Figure S7B).

### Necrosis area statistical analysis

Regarding necrosis area % in Glera, Omega thin grafting showed the lowest value (9.7 ± 1.3) compared to the W&T and FC of the same caliber. Considering the thick caliber, the necrosis areas were not significantly different with value comparable to those observed in both W&T and FC thin (Table 7).

**TABLE 7:** Percentage of necrosis was estimated by comparing two caliber ranges of propagation material: “thin grafted vines” and “thick grafted vines.”

Caliber	Grafting type	Necrosis (%)
	Omega	9.7 ± 1.3a
Thin	W&T	31.4 ± 4.8b
	FC	30.9 ± 12.6b
	Omega	31.8 ± 5.4b
Thick	W&T	27.5 ± 2.1b
	FC	15.1 ± 4.1ab

**Two-way ANOVA**	**Df**	***F*-value**

Cultivars	2	11.434***
Grafting type	2	7.405***
Cultivars x Grafting type	4	0.737^ns^

Percentages were transformed by arcosin function and normal distribution verified before two-way ANOVA.

***P < 0.001. ns, not significant.

### Physiological measurements

Results are presented according to the applied bench grafting method (FC, W&T, O) and both caliber ranges (thin and thick) of Glera grafted on Kober 5BB. Few grafted cuttings were discarded after callusing: two Omega grafted cuttings for both caliber ranges did not present complete callus; one thin W&T grafted cutting with uncomplete and hypertrophic callus; as well as four thin FC grafted cuttings. In general, thick grafted cuttings showed a more homogeneous callus although more hypertrophic than thin grafted cuttings. The callus of FC grafted cuttings was less complete and hypertrophic in both caliber ranges than in the other conditions.

#### Hydraulic traits

The effects of the caliber ranges (thin and thick) on hydraulic traits of Glera grafted cuttings were reported in Table 8. The VD as well as Dh and *K*st calculated on the 6th internode was similar between grafting types showing a negligible effect of the size of scion on the xylem morphology and hydraulic efficiency at the end of growing season.

**TABLE 8:** Mean value and standard deviation for morphological traits comparing two caliber ranges of propagation material: “thin grafted vines” and “thick grafted vines.”

Caliber	Grafting type	Vd (mm^–2^)	Dh (μ m)	*K*st (Kg Mpa^–1^ s^–1^ m^–1^)
	W&T	74.5 ± 12.3	73.1 ± 2.2	30.7 ± 8.5
Thin	Omega	78.8 ± 14.5	74.4 ± 1.2	47.7 ± 15.9
	FC	81.2 ± 7.4	76 ± 1.9	44.4 ± 9.6
	W&T	76.5 ± 13.2	74.6 ± 1.2	46.1 ± 7.1
Thick	Omega	81.1 ± 19.1	83.2 ± 2	42.2 ± 9.9
	FC	78.4 ± 20	75.5 ± 1.7	48.4 ± 19.9

**Two-way ANOVA**	**Df**		***F*-value**	

Cultivars	2	0.803^ns^	0.756^ns^	0.448^ns^
Grafting type	2	0.967^ns^	0.741^ns^	0.385^ns^
Cultivars × Grafting type	4	0.914^ns^	0.909^ns^	0.271^ns^

ns, not significant.

## Discussion

The experiments here reported aimed to investigate on possible changes induced on anatomical and hydraulic traits of just grafted vines by applying three different grafting methods on three different cvs. Field observations by Mary et al. (2017) suggest a direct relationship between foliar symptoms incidence in Esca complex over the years and the grafting method applied. It is also well known that the GTDs can not only be due to colonization and necrosis of wood tissue, but can also have strong implications of physiological disfunctions leading to the development of characteristic foliar symptoms as in diseases of the Esca complex (Lecomte et al., 2012, 2018; Claverie et al., 2020) or in declines in young vineyards.

### Anatomical observations

It is well known that after intimate contact between bionts, a new parenchymatous tissue occurs as a common response to wounding (Moore and Walker, 1981a,b; Hartmann et al., 2002; Pina and Errea, 2005). One of the most relevant aims of this study was to evaluate which bionts tissue, i.e., parenchymatic rays and cambium, contributed to the callogenesis. Failure in establishing the connection is highlighted by the presence of necrotic tissue. The ongoing of necrosis indicates that an insufficient amount of nutrients is able to reach the area where the necrotic area forms (Balbi et al., 2019), or that stress signaling originates in the area inducing the formation of necrotic cells. Necrotic cells are characterized by shrunken nucleus and cytoplasm, so producing a more stained protoplast that can be recognized in healthier tissue due to its darker appearance (Papini, 2018). This investigation was conducted studying grafting connection moving forward slide-by-slide from the rhytidome to the inner part to have a consistent evaluation of callogenesis similar to 3D x-ray tomography but with the added value of classical histology (Fernandez et al., 2022). Classical histology allows to obtain more detailed information on the composition of the tissue by differential staining. In this way, comparing the three cvs at T1, we were able to observe interesting differences in either R or S tissues, involved in the meristematic activity. In our opinion, such differences in tissue reaction could be attributed only to the cv. effect since the rootstock was the same. In particular, it is the first time, to our knowledge that Teroldego cv. has been reported to exhibit a slow process of wounding repairing. Nevertheless, Teroldego cv. grafted on Kober 5BB has showed a successful union as previously described by Prodorutti et al. (2009).

Considering the impact of the three grafting techniques on the successful connection between bionts at T3, Omega grafting still showed high distance (about 2 mm) between bionts and the connection was guaranteed by a differentiated callus filling this space, confirming the essential role of this tissue during the grafting union formation (Gautier et al., 2019). This was not considered as a critical parameter by Milien et al. (2012) since spaces between S and R were evident in both “bad” and “good graft.”

Furthermore, the results agreed with the histological observations on autografts of *Prunus domestica* as well as heterografts of *Prunus cerasus* cvs where cell differentiation and xylogenesis led to the formation of vascular connections (Gebhardt and Goldbach, 1988). At T3, the delayed response in callus differentiation was confirmed in Teroldego, which still showed meristemoids.

To date, another important parameter obtained from our histological observations was the % of necrosis areas on the graft union. From these results, it has to be noted that Omega grafting in all cvs showed the lowest % of necrotized area, revealing the best capability of connection between the two bionts at T3 (Milien et al., 2012). Furthermore, this phenomenon was confirmed by anatomical observations, where Omega grafting, independently of the cvs showed the most homogeneous and equilibrated callus production.

Regarding caliber influence, at T1 the callogenesis was scarce and unattached unions were observed in most of the grafting while at T3, Omega thin (Supplementary Figure S8C) grafting showed the best connection compared to the other grafting where no necrotized callus was observed. Furthermore, in this case, the % of the necrotized area in S and R was significantly lower than in the other samples. Omega thick grafting compared to the thin grafting showed a delayed response since the callus still exhibited meristemoids (Supplementary Figure S8B). For these reasons, only for Omega grafting we can assume that caliber of the bionts influenced callus formation.

### Hydraulic traits and vessel density

Hydraulic transport efficiency of the xylem is one of the most important traits determining the growth performance of a species as well as its capacity to respond to environmental constrains. The results of trial 1 showed that the vessel density (VD) expressed as number of vessels: xylem area ratio (excluding phloem and ray parenchyma) as well as the total vessel area: xylem area ratio (VA_f_) were significantly influenced by cultivar (*P* < 0.0001). However, the significant interaction cultivars x grafting type (*P* = 0.03) demonstrated that the grafting type had different effect within each cultivar. The *post hoc* analyses showed that combination of Glera-Omega and Cabernet Sauvignon-Full Cleft determined significant changes in VD (137 vs. 85.2 number of vessels per mm^–2^ of xylem area, *P* = 0.007). The effect of cultivars on the xylem morphology was already reported in grapevine (Pouzoulet et al., 2014). On the whole, Cabernet Sauvignon showed the lowest VD and VA_f_ in comparison to Teroldego and Glera cvs, whilst no differences were detected for Dh and Kst. These results highlighted the main effect of the genotype over the grafting type on the xylem morphology and the negligible effects of these factors on the hydraulic traits. The mean VD ranged between 87 and 140 vessels mm^–2^ and these values were higher than those reported for the same cvs in other works (Pouzoulet et al., 2013). However, two considerations have to be considered: first, we excluded the phloem and ray parenchyma areas in the calculation of xylem area; second, we sampled the internodes in the distal part of the stem and a general increase of VD from the base to the apex was reported in 8-year-old *V. vinifera* cv. Chardonnay plants (Sun et al., 2006) and cv. Nebbiolo (Schubert et al., 1999). Thus, our results were similar to those reported by Lovisolo et al. (2002), who found 100 vessels per mm^–2^ in apical stem of 2-year-old plants of *V. vinifera* L. cv. Pinot noir, grafted on *V. riparia* × *berlandieri* ‘Kober 5BB’. The higher VA_f_ recorded in Glera was determined by a higher vessels area rather than a low xylem surface (data not shown). With the same xylem area, the high vessel surface could determine a reduction of wood basal density as already reported in other woody species (Giovannelli et al., 2019). These results confirmed that VA_f_ is strongly dependent on genotype (Haworth et al., 2017). The significant effects of the cultivar on VD and VA_f_ were not found when Dh and Kst values were calculated. At a glance, this result appears somewhat counterintuitive, as, with the same xylem area, cultivar with low VD (Cabernet Sauvignon) should have wider Dh than cvs with high VD (Teroldego and Glera). It is well known that larger vessels assure higher hydraulic efficiency (lower resistance to water flow) than smaller ones (Tyree and Sperry, 1988). Conversely, the similarity of Dh values recorded in different cultivars could be due to different distribution of vessel elements in diameter classes as already reported in *V. vinifera* cvs (Pouzoulet et al., 2019). This statement can be partially supported by the significant negative correlation between VD and Dh (–0.74, *P* = 0.02) considering the three cvs pooled.

Our results showed that grafting type did not induce significant changes in vessel density and hydraulic traits. In this experiment, Glera plants had lower VD than what recorded in the first trial (in average 115 vs. 78 vessels per mm^–2^). This discrepancy in the results can be explained by the sampling of internode position along the stem. In fact, in this trial we sampled the 6th internode, while in the first trial we sampled the 10th internode in the apical part of the stem.

### Plant growth

The estimation of the parameters of the Logistic curves related to the number of internodes and plant height allowed to compare the growth trend for all the cultivars × grafting type combinations during the trial 1. Our results showed that the intrinsic growth rate (*r*, i.e., maximum growth rate) was affected by cultivars type for internode numbers and by grafting type for plant height. Intrinsic growth rate is an important parameter for many ecological applications, such as population risk assessment and harvest management (Dillingham et al., 2016) and represents the maximum potential exponential growth rate that a population can achieve under optimal resource conditions availability in its environment (Sibly and Hone, 2002). Thus, Cabernet Sauvignon and Teroldego showed higher *K* (maximum final plant height) as consequences of their high *r*. The significant effect of grafting type on *r*, supported the hypothesis that grafting type was able to modulate stem growth in grapevine. In particular, Omega type grafting determined a significant higher *r* than W&T and FC for the plant height. Contrary to our expectations, the high *r* did not correspond to final taller plants. This discrepancy could be explained by the presence of time lag in growth in Omega grafted plants that we were not able to detect for the longer timing range between measurements. These results emphasized the possible effect of the grafting type on the plant phenology.

## Conclusion

This study confirms that all the investigated grafting techniques potentially expose the conductive elements to the development of less functional tissues. The data obtained during the first vegetative season in the nursery are not enough to justify strong differences in the development of foliar symptoms in vines that had been grafted with different methods, as suggested by Mary et al. (2017). Nevertheless, we see that grafting methods induce different changes in the formation of the vascular tissue and its hydraulic traits. Grafting showed to have an impact on anatomical and functional disorders leading to necrotic tissues in the graft union. These tissues can become weak points favoring wood pathogens colonization or latent infections.

This study confirms that all the investigated grafting techniques expose potentially the conductive elements to the development of a less functional or even necrotic area which could be related to the anatomical disorders or even to the colonization by the pathogens associated to GTDs. On the other hand, the influence of the vine training and pruning on sap flow (Lecomte et al., 2012) was already suggested to strongly influence the development of the GLSD foliar symptoms (Lecomte et al., 2018).

Furthermore, by understanding the impact of three grafting techniques on the vascularisation of the grafted tissues, the present research has highlighted the timing of callus differentiation in vascular elements as the key factor to evaluate the grafting quality. The anatomical observations have revealed that the mechanical (Omega) and semi-mechanical (Full Cleft) grafting methods have a faster callusing response while the manual technique (Whip and Tongue) has a slower but greater vascularisation of the differentiated callus, especially in the first growing time, immediately after callusing, which was found to be the most critical stage.

The grafting methods and the grapevine cultivars studied were confirmed to impact the scion hydraulic traits and growth during the first vegetative season, independently from the anatomical differences initially detected after callusing. Considering that a stem expressing extensive hydraulic failure could be more prone to express GTDs related symptoms in vineyard or, in the worst cases, to die (Bortolami et al., 2021), the detected hydraulic traits in stem scion, might have a role in expressing Esca complex leaf stripe symptoms in the mature vines, potentially contributing to vine death.

In this view, the results suggest to extend the study on the same propagation material planted in vineyard, throw mid-term observations in the mature and productive vines, in order to verify the relationship between the anatomical characters and the hydraulic traits with the young grapevine decline and GTDs incidence.

## Data availability statement

The raw data supporting the conclusions of this article will be made available by the authors, without undue reservation.

## Author contributions

EB and SF: conceptualization, methodology, formal analysis, investigation, data curation, and writing – review and editing. AG and SS: conceptualization, resources, methodology, formal analysis, investigation, data curation, and writing – review and editing. CT: methodology, formal analysis, investigation, and data curation. RP: investigation and data curation. AP: data curation, resources, and writing – review and editing. SD: conceptualization, methodology, and supervision. LM: conceptualization, methodology, resources, and writing – original draft, review and editing, supervision, project administration, and funding acquisition. All authors contributed to the article and approved the submitted version.
