# Supplementary material for: Xylem anatomy and hydraulic traits in Vitis grafted cuttings in view of their impact on the young grapevine decline
Source: Front Plant Sci. 2022 Oct 5;13:1006835. doi: 10.3389/fpls.2022.1006835 (PMC9581319; doi:10.3389/fpls.2022.1006835)
Supplement: Supplementary file 1 [file Data_Sheet_1.docx]

Supplementary Materials

Xylem anatomy and hydraulic traits in *Vitis* grafted cuttings in view of their impact on the grapevine young decline

Enrico Battiston^1†^, Sara Falsini^2†^, Alessio Giovannelli^3^, Silvia Schiff^2^, Corrado Tani^2^, Roberta Panaiia^2^, Alessio Papini^2^, Stefano Di Marco^4^ and Laura Mugnai^1^

^†^ These authors have contributed equally to this work and share first authorship

^1^ Università degli Studi di Firenze, Dipartimento di Scienze e Tecnologie Agrarie, Alimentari, Ambientali e Forestali – Sezione Patologia Vegetale ed Entomologia, P.le delle Cascine 28, 50144 Firenze, Italy

^2^ Università degli Studi di Firenze, Dipartimento di Biologia – Laboratorio di Biomorfologie, Via Micheli 3 - 50121 Firenze, Italy

^3^ Consiglio Nazionale delle Ricerche, Istituto di Ricerca sugli Ecosistemi Terrestri, Via Madonna del Piano 10, 50019 Sesto Fiorentino, Italy

^4^ Consiglio Nazionale delle Ricerche, Istituto per la Bioeconomia, Via P. Gobetti 101, 40129 Bologna, Italy

**Anatomical study**

**Grafting trial 1**

**Glera scion onto K5BB rootstock:**


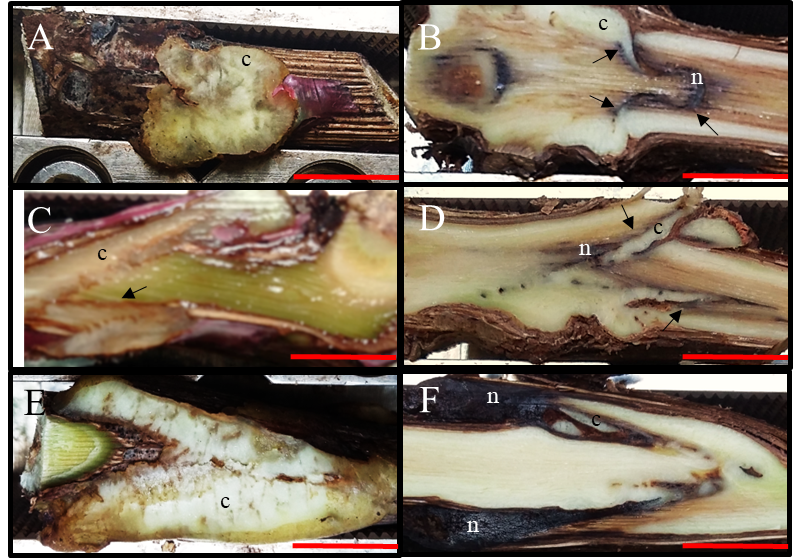


**Figure S1**: Photographs of graft longitudinal sections of Glera scion onto K5BB rootstock, at T1 (A, C, E) and T3 (B, D, F). Callus formation in different graft union shapes: Omega (A, B), W&T (C, D) and FC (E, F). Scale Bar: 0.8mm. c= callus; n= necrosis; black arrows= discontinuity.


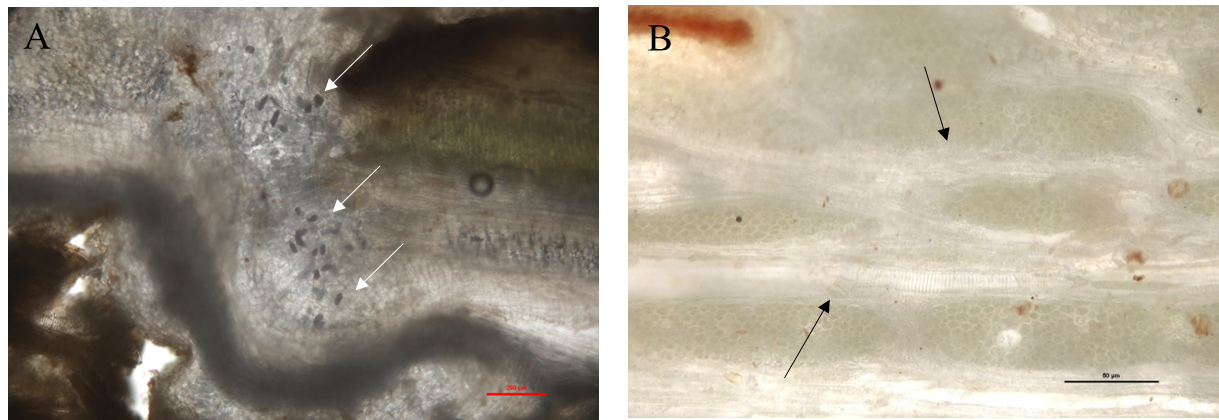


**Figure S2**: LM images of longitudinal sections of Glera grafting. (A) Not structured callus with Calcium Oxalate crystals (arrows) in FC grafting at T1. (B) Structured callus with xylem vessels (arrows) in W&T grafting at T3.

**Cabernet Sauvignon scion onto K5BB:**


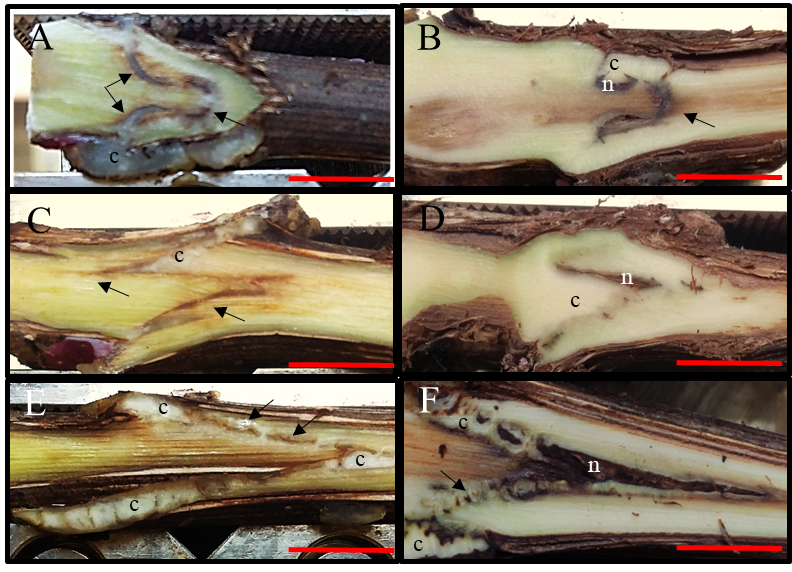


**Figure S3**: Photographs of graft longitudinal sections of Cabernet Sauvignon scion onto K5BB rootstock, at T1 (A, C, E) and T3 (B, D, F). Callus formation in different graft union shapes: Omega (A, B), W&T (C, D) and FC (E, F). Scale Bar: 0.8mm. c= callus; n= necrosis; black arrows= discontinuity.


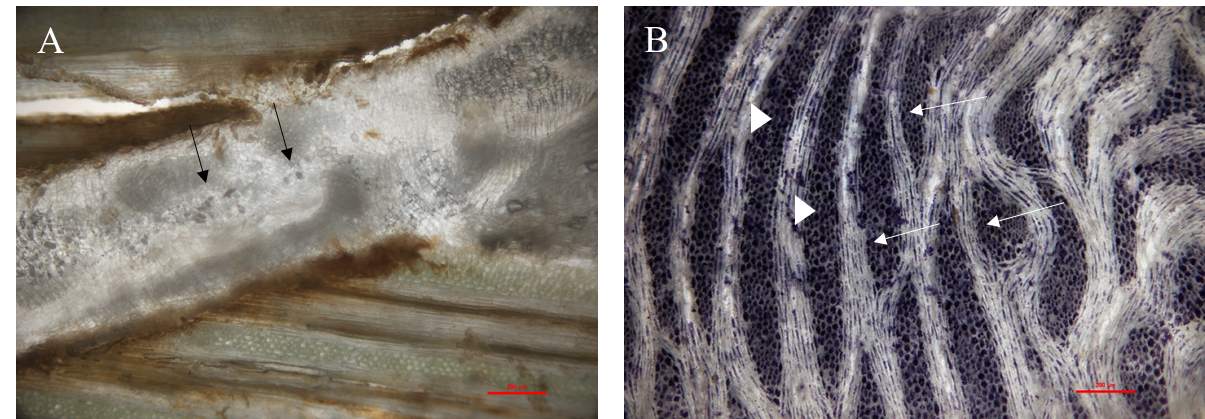


**Figure S4**: LM images of longitudinal sections of Cabernet Sauvignon grafting. (A) Not structured callus with Calcium Oxalate crystals in W&T grafting at T1. (B) Structured callus with xylem vessels (arrows) among parenchymatous tissue rich in starch (head arrows) at T3 (Lugol staining).

**Teroldego scion onto K5BB rootstock:**


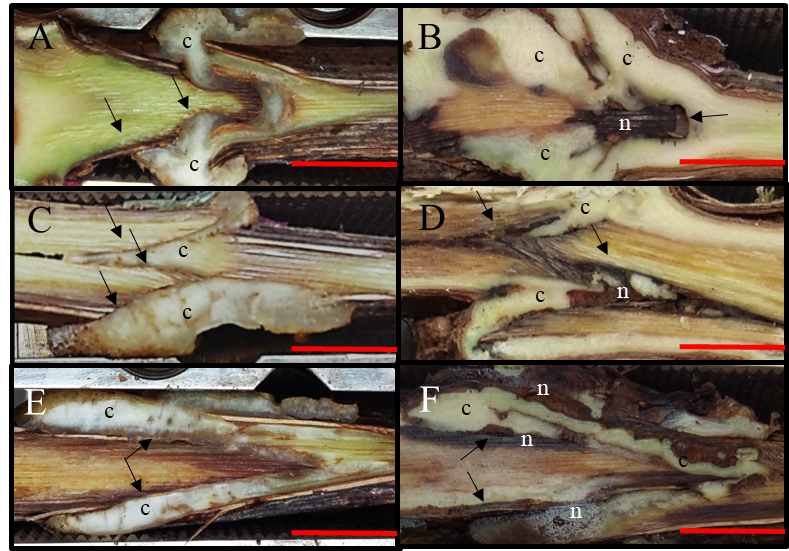


**Figure S5**: Photographs of graft longitudinal sections of Teroldego scion onto K5BB rootstock, at T1 (A, C, E) and T3 (B, D, F). Callus formation in different graft union shapes: Omega (A, B), W&T (C, D) and FC (E, F). Scale Bar: 0.8mm. c= callus; n= necrosis; black arrows= discontinuity.


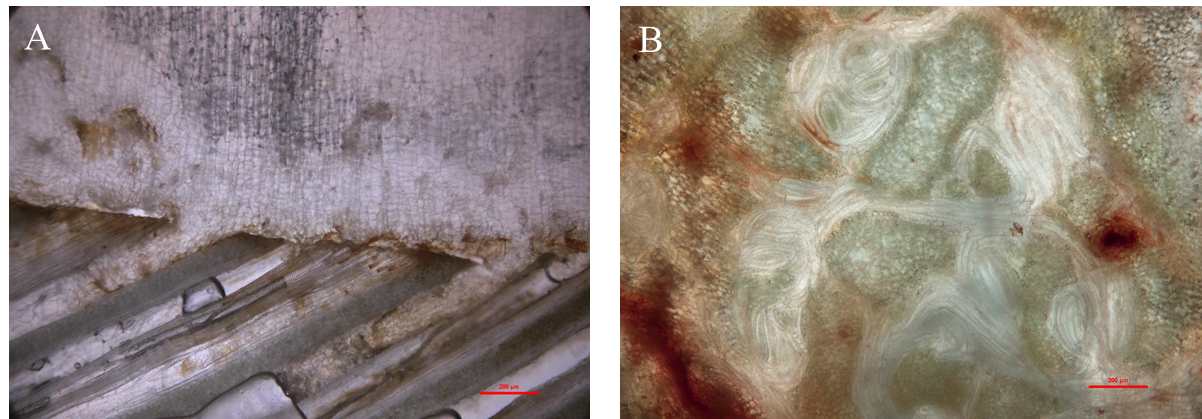


**Figure S6**: LM images of longitudinal sections of Teroldego cv. (A) Not structured callus in Omega grafting at T1. (B) Callus in differentiation characterized by meristemoids.

**Grafting trial 2:**


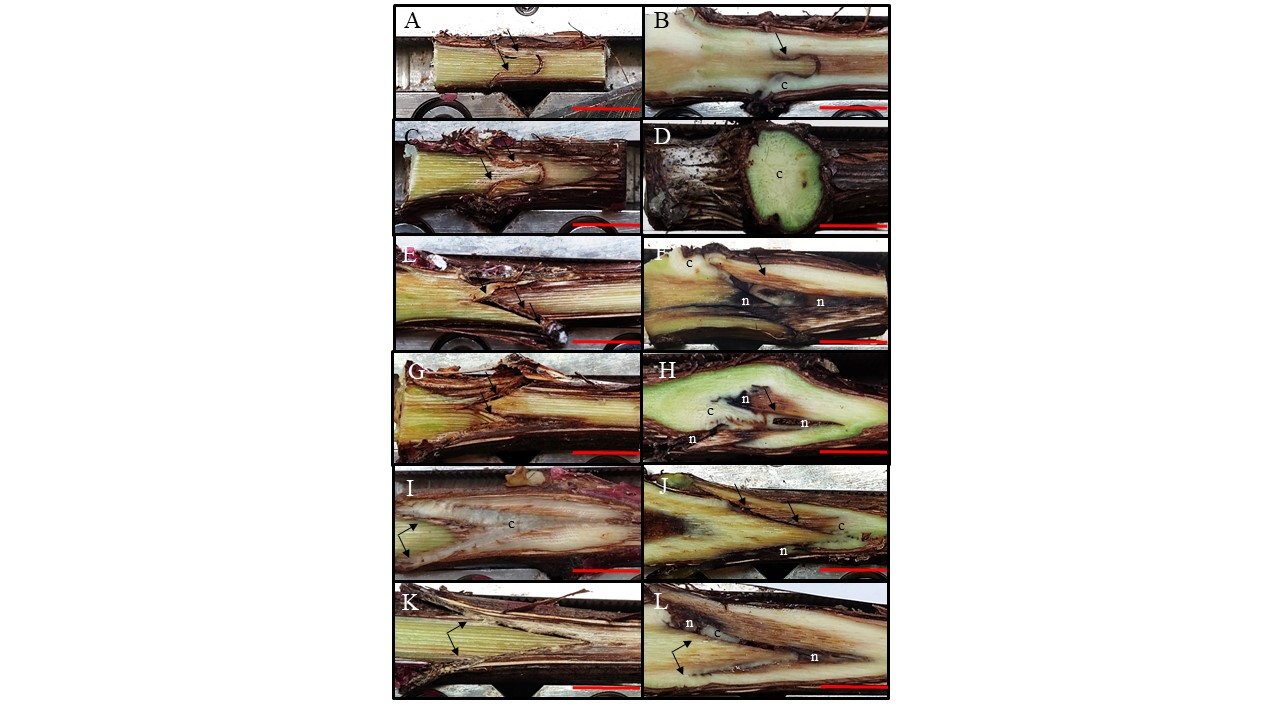


**Figure S7**: Photographs of graft longitudinal sections of Glera scion onto K5BB rootstock, at T1 (A, C, E, G, I, K) and T3 (B, D, F, H, J, L). Callus formation in different graft union shapes: Omega thin (A, B), Omega thick (C, D), W&T thin (E, F), W&T thick (G, H), Full Cleft thin (I, J) and Full cleft thick (K, L). Scale Bar: 0.8mm. c= callus; n= necrosis; black arrows= discontinuity.


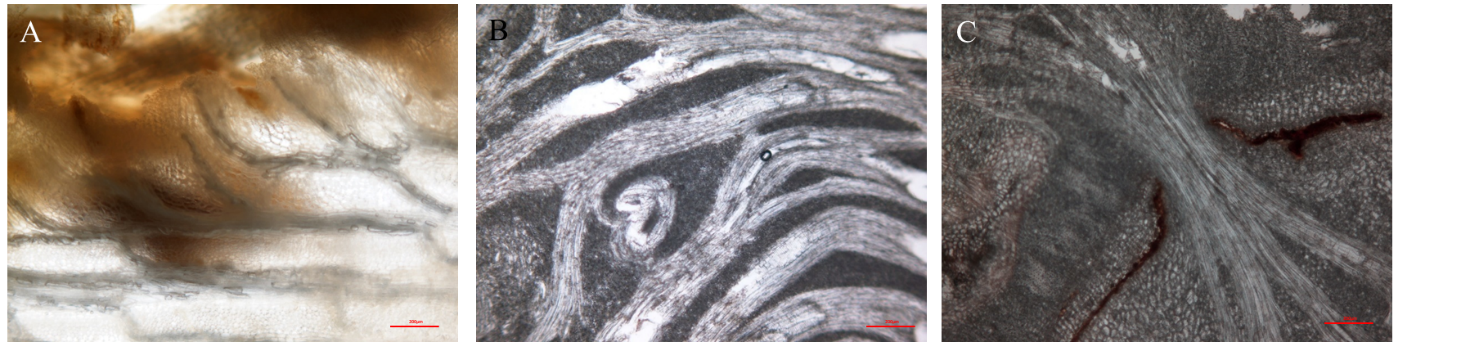


**Figure S8**: LM images of longitudinal sections of Glera grafting. (A) Differentiated Callus in FC thin grafting at T1. (B) Callus in differentiation characterized by meristemoids in Omega thick and (C) differentiated callus in Omega thin at T3.

**Table S1**: Parametrization of number of internodes by Logistic model.

| **Cultivar** | **Grafting type** | | **Logistic model** | |
| --- | --- | --- | --- | --- |
|  |  | *K* | y_0_ | r |
|  |  |  |  |  |
| Glera | omega | 21.5±3.9d | 2.6*10^-4^ | 0.058±0.008 |
|  | FC | 24±4.9cd | 4.2*10^-4^ | 0.057±0.008 |
|  | W&T | 22.8±5.8cd | 1.4*10^-3^ | 0.053±0.01 |
| Teroldego | Omega | 23.7±5.3cd | 8.5*10^-5^ | 0.063±0.01 |
|  | FC | 27.0±4.8bcd | 1.4*10^-4^ | 0.062±0.008 |
|  | W&T | 26.7±4.3bcd | 4.5*10^-4^ | 0.064±0.004 |
| Cabernet Sauvignon | Omega | 33.0±5.0a | 5.2*10^-4^ | 0.061±0.009 |
|  | FC | 30.5±4.1ab | 6.6*10^-5^ | 0.063±0.005 |
|  | W&T | 27.2±4.4bc | 7.1*10^-5^ | 0.063±0.006 |
|  |  |  |  |  |
| **Two-way ANOVA** |  |  |  |  |
| **Factors** | **Df** |  | ***F value*** |  |
|  |  |  |  |  |
| Cultivars | 2 | 28.128*** | 3.811* | 10.873*** |
| Grafting type | 2 | 1.334 ^ns^ | 0.877^ns^ | 0.08^ns^ |
| Cultivars x Grafting type | 4 | 3.78* | 2.398^ns^ | 0.911^ns^ |

*P < 0.05, ***P < 0.001, ns, non significant.
